# The Treatment of Fractures

**Published:** 1904-09-03

**Authors:** 


### THE TREATMENT OF FRACTURES.

DR. M. W. WARE[1] calls attention to several important points in connection with the treatment of fractures. In the first place he strongly deprecates any attempts to elicit crepitus in a suspected case, except with the patient under anæsthesia. His reasons are that crepitus is accompanied with severe pain, while the presence of a fracture can usually be recognised without its aid and especially if the *x* rays be used. An important sign of fracture is localised tenderness, and this should be determined by the gentlest palpation, and it should then be determined whether pressure applied to the ends of the bone causes pain to be felt in the region of local tenderness already observed. As anæsthesia is accompanied by certain risks, he does not recommend its use except in fractures in the neighbourhood of joints, and where there is much displacement. Where an anæsthetic is necessary he prefers to use ethyl chloride. In the treatment of a fracture the question often arises, what ought to be done at first. Dr. Ware's advice is to do nothing until the most suitable device can be applied. At most it may be desirable in the meantime to apply a bandage with moderate pressure to reduce the swelling. There is a general tendency, he thinks, to overtreat fractures; and he states that in the case of the upper extremity the callus is sufficiently firm at the end of two weeks to keep the ends of the fractured bone in place, so that infant, youth, or adult should, in most instances, be without splints at the expiration of this time, and the simplest of active (not passive) movements should then be allowed. The chief aim should be the quick restoration of function rather than a perfect cosmetic result, especially in joint fractures. Prognosis should be very guarded even in the most favourable cases, as untoward results can never be excluded with certainty. With regard to apparatus Dr. Ware condemns the use of any ready-made splints except in fractures of the thigh. The simplest materials are the best, *e g.* plaster of Paris, pasteboard, cigar-box wood, and non-absorbent cotton.

[1] Medical News, Aug. 13.

